# Some Points in Incipient Orthopedic Cases1An address before the last meeting of the Niagara College Alumni Association.

**Published:** 1899-05

**Authors:** Marcell Hartwig

**Affiliations:** Buffalo, N. Y.


### SOME POINTS IN INCIPIENT ORTHOPEDIC CASES.[1]

By MARCELL HARTWIG, M. D., Buffalo, N. Y.

IN A SOCIETY as erudite as I have the honor to address, some things I have to say may appear trite; still it pays to take occasionally a bird's-eye-view of well-known objects. Here and there they will appear in a new and instructive attitude.

It has come to pass, and that by no means seldom, that I have encountered cases in practice which I have found lamentably progressed for the worse by early neglect or want of diagnosis, and so certainly have many of you.

Let us be veracious and say how often have we examined a newborn with that careful attention which would make sure of not having overlooked some *vitium primæ formations?*

Do we not rely too much upon nurses and aunts, glad of being through with the martyrdom of having listened to the groans of the laboring woman and of having alleviated them as best we could, and happy of having found the time to recuperate our drooping spirits in the rest of well-earned slumber? Still we should not do so, but give the newborn a good and thorough investigation. For many a defect unobserved may develop into a state greatly to be deplored where we could have helped so easily. Once in awhile the diagnosis may be difficult. Thus let me divide the subject for practical purposes into diagnosis and treatment, because it will answer as well for later troubles. Of course, it is not my province to tell you how to diagnosticate the commonly so-called orthopedic cases, but permit me only to make a few remarks about the difficult ones.

Here the safest rule is like with a continued fever where a diagnosis is impossible—to take and treat it for the worst possibility within the range of probabilities. If we have a patient, who complains about some pain in the knee and he shows some indistinct lameness without a swelling of the knee, do not give him a liniment, especially if it is a child—make it a hip disease—put the patient in a plaster cast or Taylor's splint. If you have erred and it was a sciatica or rheumatism of the hip-joint you have not to blame yourself of not having provided for the worst possibility without having played the cheap, smart gag of intimidating the patient in order to appear afterwards as the archangel who killed the dragon. I know, this contemptible way of making a reputation is beneath the dignity of a Niagara Alumnus. But to be a little systematic. The most difficult diagnosis

1. An address before the last meeting of the Niagara College Alumni Association.

in the newborn is the recognition of hip-joint dislocation. Here I can give one advice only. See if the Nelaton line falls correctly and in case of doubt take an x-ray photo. In fact I am not aware that this defect was ever recognised in the newborn.

Lorenz's method of reduction has made the cure of this hideous defect so beautifully easy, and grows so steadily more and more in difficulty as age advances, that we ought to make every endeavor to recognise the defect before walking or standing has aggravated it. The capsule ought not to be contracted—yet according to all we know of the pathology of this defect, and so early recognition is urgently desirable. I hope one of you will be the first to publish the first case in the newborn to the glory of the Alumni Association and of the Alma Mater.

The club-feet of the newborn are easy of diagnosis, but attention is necessary, or else light degrees of pes planus or varus will be overlooked simply at a time when correction is marvelously easy. Hernia of the navel is once in awhile difficult to diagnosticate, as simple as it would seem it ought to be, but sometimes the folds of the skin are so prominent as to simulate hernia. The safest is the rule given before—treat as though the worst possibility existed.

How many of us look for an undescended testicle in the newborn and yet we should, as its consequences are often troublesome or worse, and a truss with its pad above it will invariably force it down into position. Let us not forget to look for hydrocele, because the cure of it is so easy at the time without operation. Hernia likewise.

The majority of other defects in the newborn, birthmarks, closures, clefts, defective or multiple fingers, web-fingers and the like, are easy of diagnosis and are seldom overlooked by attendants, but as a minimum ought to be inquired about, as the treatment, respectively, degree of surgical interference, grows with every day of life, and as the flesh of the newborn heals remarkably readily. If anything is worthy of mention in the line of diagnosis it is the Erbs (imperial) paralysis. Light degrees may be overlooked, but are probably cured by the *ars medicatrix naturæ*; so may the light cases of anterior poliomyelitis later on. Finally, fractures occur in utero and may be healed at the time of labor in a wrong position. If we make any during version, we ought to freely acknowledge the fact after hearing respectively, feeling the ominous click and ascertaining the fracture.

In later life the difficult diagnosis in orthopedics (a word by the way already used by Andry in 1741) was illustrated already concerning the hip, and so it remains everywhere at the very beginning of trouble and here the first and last rule is examine! examine! measure, move, compare right and left and again where in doubt treat for the worst possibility in the range of probabilities. If we do not forget the very first symptoms of orthopedic affections, we will be alert to recognise incipient disease and obtain corresponding good results. For this purpose the first rule is to strip the patient to Adamitic appearance. A stiff bearing of the trunk is suspicious of Pott's disease, particularly if accompanied by shooting pain in the chest or abdomen in the night. An unduly prominent os naviculare shows beginning flat-foot, abduction and outward rotation of the leg, beginning hip-disease, particularly when accompanied by night cries. A raised shoulder or projecting lower angle of the scapula point to lateral curvature, particularly if accompanied by night cries of pain. An unduly prominent abdomen speaks for rickets; if abdominal pain is complained of, it may be Pott's disease after all. Walking nakedly will show you at once genu valgum or varum. The lightest limp will be revealed, which may be readily overlooked when the child is clothed or partially clothed. Genu recurvatum may escape your notice, if you examine the one leg alone, but never when the child is stripped. So it is, even with a hernia. The rule for diagnosis everywhere is to observe—but how can we observe, when the child is clad—there we observe the tailor's art chiefly.

Sudden upstarts and yelling from pain in the night are suspicious of tubercular disease, if the pain is in the chest or abdomen and no reason found there of Pott's disease; if the pain is in the knee, of hip-disease, if configuration of the knee and its mobility are perfect. If local changes in the knee are noticeable, of course, the knee itself, is the seat of the malady. Pain in the foot, without swelling, is often due to developing of flat-foot, while the congenital form is painless.

Muscular spasm, if not due to spastic paralysis, or other nerve disease, points to tubercular joint affection, sometimes, though, to nontubercular troubles. Here the thermometer may reveal the nature of the trouble—meanwhile consider it tubercular. In drop-wrist, do not forget lead-poisoning. Crying of a baby when being lifted, dressed and the like, requires the most painstaking examination. Laying it upon the abdomen may reveal Pott's disease by an undue prominence of a processus spinosus, while the sitting posture may not, because the prominence is hidden in the general bending of the back.

*Treatment.*—As everywhere the scientific conception of the nature of the disease, *i. e.*, the pathogeny is the keystone to success in treatment. You know it is too vast a field to cover in an essay like this and that I propose to mention some smaller points only here and there.

Beautiful results can be obtained with manipulation in defects of the newborn. Considerable degrees of pes planus or varus are entirely curable by daily and several times daily stretching the contracted tissues and correcting the faulty position. All that is needed is to teach the mothers or attendants how to do it—the essential points—and to convince ourselves that our advice is properly executed. This last point is the most important, as the heart of a mother bleeds each time when she should make her little darling or picanniny suffer pain; even we do not feel very pleasant in fulfilling our duty by applying the strong manipulation required. But success will follow even in some cases where two months later operation would become the *sine qua non.*

Concerning the flat-foot in later years Whitney plates alone are not always sufficient. In severe cases correction in narcosi and maintenance in the corrected position by a plaster of Paris cast for a week or so before making the plates shortens the cure materially.

In slight cases of varus tenotomy of the Achilles tendon suffices, but hardly ever without after-treatment. So do not neglect to keep up the control for months. This maintaining of control is most important with the poor, who naturally avoid it on account of expense. Therefore you will always do wisely to inform the parents at the outset that you shirk responsibility unless the child is presented to you at reasonable intervals for a year, let the case be as slight as it may. The same rule should apply to other tenotomies. A woman blames me for her child's persisting wry neck, where I ought to blame her had I not neglected the above injunction. Take again the severe cases of varus and Phelps' operation, which is a truly wonderful and daring application of modern asepsis; do not forget that the over correction in the plaster cast after the operation should not be an over correction by force. The cutting should be sufficient to admit over correction without force, or else you may see a few toes slough away. I have cognisance of such a case.

Here it may not be amiss to say a few words about plaster casts in general. This exquisitely important aid in orthopedics cannot be mastered unless by frequent use. The ready-made bandages on the market are generally good material, only for Sayres's jacket they are too narrow. We ought to have six or eight inch wide bandages for rapid work. Some experience a difficulty in maintaining certain positions, because a change of hands is needed. Here the most simple expedient is a flannel extension bandage, which after it has done its duty is cut off and remains inside of the cast. Concerning tendons and bones, do not forget that a very slanting incision permits stretching with immediate suture. Only today I saw in a report from Vienna—*Medical News*, April 23, 1898, page 538—stating in a manner which implies a novelty in it, that Frank cured thus an equinus, while I have proposed this method long ago for bones and tendons in several publications.

Hernia and hydrocele are two affections which are mostly recognised by attendants as undue defects of the newly born, still it will do no harm if we ourselves in our daily visits during the lying-in period pay some attention to the possibility of these congenital defects, as at that time the natural formative tendencies are so much in our favor, that slight attention is apt to produce a cure with the simpler means. It seems not to be very widely known that hydrocele of the newborn can be cured by expression. If we consider the pathogeny it is natural. The hydrocele of the newborn is simply an open connection of the peritoneal cavity with the tunica vaginalis cavity, the tunica vaginalis being in reality peritoneum, the liquid is peritoneal serum. By slow and steady compression we force the liquid into the peritoneal cavity and the hydrocele is cured, but if the connecting canal is exceedingly wide we may have to do it again. As the natural tendency is to contraction and agglutination of the serous tube, the cure is permanent. Of course, where a steady pressure of a minute or two shows no softening of the hydrocele, the communication is closed already—we are too late and operative measures are needed. The same natural tendency to contraction permits a rapid cure in hernias by the pressure of trusses, but very careful personal attention of ours is needed. If crying with an ill-fitting truss makes the hernia come down once, we have lost a week and if twice perhaps a month and a few times oftener and we can as well go for Mr. Bassini. The treatment is altogether not as important as the diagnosis, because the latter contains within itself the elements of cure. I am perfectly certain of having obtained absolute cures of white swelling of the knee, of Pott's disease and of hip-joint disease by earnest, early attendance, meaning by absolute, conditions obtained entirely indistinguishable from the normal. The means of cure were, of course, immobilisation, extension and attention to the general health. If we survey the miserable results of operative treatment in tubercular diseases, we cannot urge the parents forcibly enough to have all suspicious cases treated at once as though they were the very worst. The same rule applies to rickety deformities, not so much because of their danger, as because we can save the poor innocent children future disappointments and operations.

In beginning scoliosis dextra we may obtain a cure by teaching to write with the left hand, or to sit on a sideways slanting seat and other appropriate muscular exercises, whereas later scoliosis is well-nigh incurable. In Germany they have wisely started upright writing to prevent scoliosis. The teachers should be informed to pay attention to the posture of the children on this account and to prevent myopia.

It is a state of affairs very much to be deplored that the old system of the family physician is going under with the modern spread of specialisation—the system where the family physician was paid by the year as in Europe, where he visited uncalled for, detected growing defects in their incipiency and was not under the suspicion of making a case for himself. If this is the sequel only of the vastness of the field of medical science, we can but correct it by increasing the demands for promotion, and I am sure the Niagara Alumni will be found in the front rank of those who support every measure tending to reach a maximum standard of medical education, and thus help to reconstruct the ideal physician.

884 Main Street.

